# Social Challenges on University Campuses: How Does Physical Activity Affect Social Anxiety? The Dual Roles of Loneliness and Gender

**DOI:** 10.3390/bs15081063

**Published:** 2025-08-05

**Authors:** Yuyang Nie, Wenlei Wang, Cong Liu, Tianci Wang, Fangbing Zhou, Jinchao Gao

**Affiliations:** 1 College of Physical Education and Sport, Beijing Normal University, Beijing 100875, China; 202422070104@mail.bnu.edu.cn (Y.N.); congliu@bnu.edu.cn (C.L.); 202422070144@mail.bnu.edu.cn (F.Z.); 2 College of Education for the Future, Beijing Normal University, Zhuhai 519087, China; wwl@bnu.edu.cn; 3 School of Physical Education, Ningxia University, Yinchuan 750014, China; 12024130217@stu.nxu.edu.cn

**Keywords:** physical activity, social anxiety, loneliness, mediating effect, college students

## Abstract

Social anxiety is a prevalent mental health concern among college students, often intensified by academic and interpersonal pressures on campus. This study investigated the relationship between physical activity, loneliness, and social anxiety among college students, aiming to examine the mediating role of loneliness in the process of physical activity affecting social anxiety, as well as the moderating role of gender in this mediating effect. A cross-sectional research design was adopted, and data on physical activity levels, loneliness, and social anxiety were collected through questionnaires completed by 638 students at a university in China. This study conducted a single-factor Harman test, descriptive statistical analysis, reliability analysis, correlation analysis, and independent-samples *t*-tests, and it modeled the moderated mediation effect. The results showed that physical activity was significantly and negatively correlated with both loneliness and social anxiety. Loneliness played a mediating role in the influence of physical activity on social anxiety, and this mediating effect was moderated by gender, being more pronounced in the female group. This study concluded that physical activity can help alleviate social anxiety, but the mechanism involving the reduction of loneliness is more apparent in women, indicating the need to consider gender differences when developing interventions, as there may be other, more significant reasons for men.

## 1. Introduction

Social anxiety, or social anxiety disorder (SAD), is a prevalent mental health issue that involves a chronic dread of social or performance-related circumstances where one may be assessed poorly (Guan, 2024). Patients avoid social events or experience acute anxiety due to this phobia (Andersson & Carlbring, 2010). Universities demand regular collective contact; thus, many inescapable social events improve students’ social abilities but can cause anxiety in some. Social anxiety is common among college students and negatively impacts their health, academic performance, and social life (Jesús et al., 2024). College students are more susceptible to social anxiety due to their greater interpersonal contacts and social pressure, according to many studies (Li et al., 2024; Lin & Fan, 2022). Stress is also often regarded as one of the factors contributing to early dropout (Jesús & Puerta, 2022). How to prevent college students’ social anxiety while providing collective education at colleges is a prominent issue for study.

### 1.1. Relationship Between Physical Activity and Social Anxiety

Any movement of the body made by the skeletal muscles that demands the use of energy is considered physical activity (Peters & LoBue, 2024). Neurotransmitter release, especially monoamine neurotransmitters like dopamine, serotonin, and norepinephrine, may be directly impacted by exercise (Kruk et al., 2020). The control of emotions depends on these neurotransmitters (Chen & Nakagawa, 2023). The way college students manage and experience negative emotions (such as stress, despair, and anxiety) on a daily basis is closely linked to their ability to regulate their emotions (Tsujimoto et al., 2024). Large-scale population studies and theoretical processes provide evidence of the health advantages of physical exercise. Adults who frequently participated in physical sports like long-distance cross-country skiing, for instance, had much reduced rates of long-term anxiety, according to a 21-year follow-up study of nearly 390,000 adults (Svensson et al., 2021). This implies that developing and sustaining long-term habits of physical exercise may provide anxiety protection. It is important to note that anxiety disorders, such as social anxiety and generalized anxiety disorder (GAD), can have high rates of co-morbidity. For example, Mennin et al. (2009) found that the 12-month co-morbidity rate between GAD and social anxiety may reach 47%. Regular exercise may thus be a useful strategy to lower the chance of acquiring a number of anxiety disorders, such as social anxiety and generalized anxiety, in addition to improving mood via modifying neurotransmitters.

Social information processing theory (SIC) suggests that individuals’ behavior does not occur in isolation but is profoundly influenced by the social context in which they find themselves and the information they provide (Crick & Dodge, 1994). When individuals engage in physical activity, especially in a group or socially oriented activity, the context in which they find themselves provides a range of new social information. This information may include positive feedback (e.g., peer encouragement, collaborative success), new opportunities for social interaction, and model behaviors for observing how others respond to social situations. An individual’s process of interpreting and processing this information shapes their subsequent social attitudes and behavioral patterns.

### 1.2. The Mediating Role of Loneliness

Loneliness, as an important public health issue, is closely linked to multiple mental health challenges. Although previous research has focused on older adults and appears to support the generalized view that older adults are lonelier, the reality is more complex. Younger age groups tend to show a higher prevalence of loneliness, mainly due to differentiated social needs and interaction expectations among individuals of different ages (Barreto et al., 2021). The more intense the sense of loneliness, the more severe the anxiety (Beutel et al., 2017), depression (Peerenboom et al., 2015), and psychotic symptoms (Badcock et al., 2015) are as well. Lim et al. (2016) examined whether depressive symptoms, symptoms of social anxiety, and paranoia were associated with loneliness over a 6-month period. While higher loneliness predicted more severe social anxiety, depression, and paranoia symptoms at subsequent time points, only increased social anxiety symptoms predicted sustained changes in loneliness. Thus, loneliness may be more closely linked to social anxiety than other mental health problems (Eres et al., 2023). In addition, clinical trials have provided further evidence of this link, with treatments for both loneliness and social anxiety being associated with significant reductions in the subsequent other (O’Day et al., 2021).

Furthermore, loneliness is strongly associated with physical activity. A review study suggested that physical activity helps to reduce loneliness, while loneliness itself may reduce the likelihood that individuals will engage in physical activity (Pels & Kleinert, 2016). More broadly, the mechanism by which physical activity reduces loneliness involves improving mental health and the quality of social relationships (Umberson & Karas Montez, 2010), which may reduce loneliness. Recently, a longitudinal study from Spain further confirmed the bidirectional relationship between physical activity and loneliness (Rodeiro et al., 2025). In addition, a systematic review has shown that interventions targeting physical activity have an important role in reducing loneliness (Ahn et al., 2024). Thus, based on the evidence from (O’Day et al., 2021) clinical interventions on loneliness and social anxiety, loneliness may play a mediating role in the relationship between physical activity and social anxiety. In addition, emotional regulation offers the possibility of this mediating process, as physical activity has been shown to improve individuals’ emotional regulation (Bernstein & McNally, 2018). There is a significant correlation between emotional regulation strategies and patterns and loneliness and social anxiety (Preece et al., 2021; Jazaieri et al., 2014).

### 1.3. Gender Moderation Effect

Previous empirical studies have concluded that there are differences in physical activity, loneliness, and social anxiety across genders (Azevedo et al., 2007; Barreto et al., 2021; Asher et al., 2017). Physical activity is a behavioral factor, and gender tends to play a moderating role in behavioral–cognitive outcome models constituted by physical activity (Riddervold et al., 2024; Gomez-Baya et al., 2017; Zhao et al., 2024b; Gijón Puerta et al., 2022). Thus, there may be a moderating effect of gender on physical activity, loneliness, and social anxiety.

**Figure 1:**
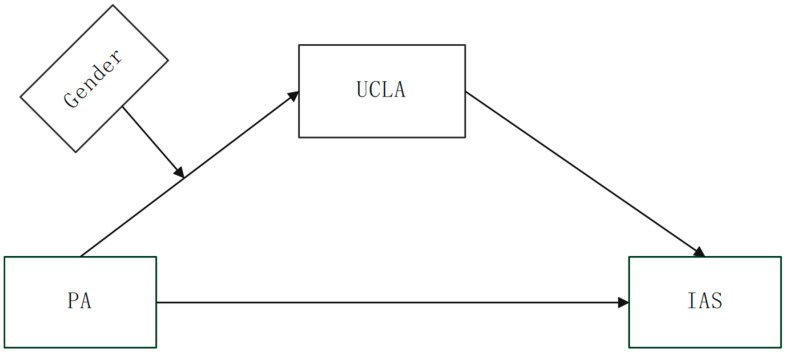
Moderated mediation model. Note: PA: physical activity; IAS: social anxiety; UCLA: loneliness.

In summary, existing research has confirmed the association between physical activity and loneliness and social anxiety among college students. Although there are previous studies on physical activity and social anxiety among college students, they are not perfect and do not involve loneliness. Therefore, the mechanisms of the association between the three need to be further explored. This study aims to fill the gaps in the existing research and deeply explore the complex relationship between physical activity, loneliness, and social anxiety. Therefore, based on the social information processing theory, this study argues that a series of cognitive and emotional processes experienced by college students while performing physical activity, which strengthens their adaptive capacity, help to reduce their loneliness and, in turn, alleviate social anxiety with a positive impact. Thus, the following hypotheses are proposed, and the corresponding model is shown in Figure 1.

**H1.**  *There is a negative correlation between physical activity and social anxiety.*

**H2.**  *There is a negative correlation between physical activity and loneliness.*

**H3.**  *Loneliness mediates the effects of physical activity on social anxiety.*

**H4.**  *Gender moderates the association between physical activity and social anxiety.*

## 2. Methods

### 2.1. Participants and Process

In March 2025, the research team conducted a cross-sectional study among undergraduate students from the first to the fourth year at the Zhaihai Campus of Beijing Normal University. All the procedures followed the Declaration of Helsinki’s Ethical Principles for Medical Research Involving Human Subjects and other applicable laws, rules, and ethical codes. To guarantee the study’s integrity, all the participating investigators underwent rigorous training before the survey. In accordance with the STROBE criteria for cross-sectional research, we developed a questionnaire that includes basic sociodemographic information, as well as measures of physical activity levels, social anxiety levels, and loneliness. The preface to the questionnaire provides a detailed description of the survey. After obtaining the students’ consent, an electronic questionnaire was distributed via WeChat. A total of 679 students responded. Subsequently, one dedicated researcher screened the submissions according to the following criteria: (1) excluding samples with missing values in the responses; (2) removing responses with homogeneity and regularity, e.g., almost the same for all the responses to a question; and (3) removing responses that were too short, e.g., less than 60 s. Finally, a total of 41 were excluded, and 638 students’ data were included, with an efficiency rate of 93.95%. As shown in Table 1, there were 372 (58.3%) males, 266 (41.7%) females, 273 (42.8%) freshmen, 181 (28.4%) sophomores, 123 (19.3%) juniors, and 61 (9.6%) seniors, and the mean age of the participants was 19.82 years (SD = 1.39).

### 2.2. Measures

#### 2.2.1. Physical Activity

In this study, the Physical Activity Rating Scale for Adolescents—3rd Edition (PARS-3) was used to assess the physical activity of college students. The scale, developed by Japanese scholar Hashimoto (2005) and revised by (Liang, 1994), measures physical activity participation using five gradients that measure intensity, duration, and frequency. The overall physical activity (PA) score is calculated by multiplying the intensity (1–5) by the time (0–4) and frequency (1–5). Thus, PA scores vary from 0 to 100. Scores are characterized as low (≤19), moderate (20–42), or high (≥43) based on the total PA scale. This scale’s Cronbach’s alpha was 0.637 in this investigation.

#### 2.2.2. Loneliness

In this study, the University of California, Los Angeles Loneliness Scale, Third Edition (UCLA) was used to assess students’ level of loneliness. The scale, revised by Russell (1996), consists of 20 self-assessment entries (e.g., “I often feel a lack of common language with those around me”) and is scored on a 4-point Likert scale (1 = never, 4 = always), with 9 of the entries being reverse-scored. The overall score is 20–80, with higher values suggesting loneliness. Among the most extensively used standardized loneliness measures, the scale has strong psychometric qualities. The scale’s Cronbach’s alpha was 0.882 in this investigation.

#### 2.2.3. Social Anxiety

The Social Interaction Anxiety Scale (IAS) measured respondents’ social anxiety in this research. The Leary (1983) measure has 15 self-assessment questions on a 5-point Likert scale (1 = not at all, 5 = fully), 4 of which are reverse-scored, with a total score ranging from 15–75, with higher scores indicating more social anxiety. The Cronbach’s alpha value for this scale was 0.883 in this research.

### 2.3. Statistical Analysis

In this study, a standardized data analysis process was used to ensure the rigor of the results. In the data preprocessing stage, the collected data were first systematically screened and cleaned. Second, this study utilized SPSS version 27 software to perform a single-factor Harman test, descriptive statistical analysis, and normality tests to determine normality by skewness and kurtosis, to test the internal consistency of the scales using Cronbach’s alpha coefficient (Tavakol & Dennick, 2011), as well as to perform Pearson correlation analyses and independent samples *t*-tests. To test models with moderated mediation effects, this study utilized models from PROCESS 4.2 in SPSS. Additionally, this study conducted simple slope analyses to test for all the potentially significant interaction effects. Considering that data standardization in mediation analyses helps to eliminate size differences, reduce multicollinearity, and enhance model interpretability and stability (Hayes & Rockwood, 2017), this study standardized the data. To enhance the robustness and precision of our analysis results, this study used the bootstrap method, set the confidence level to 95%, and performed 5000 resampling iterations to construct confidence intervals (CIs). In addition, this study used the ±1 standard deviation method to detect the effect of gender at different levels and centered all the variables. The significance of an effect was judged based on whether its 95% CI contained a zero; if the 95% CI did not contain a zero, the mediating or moderating effect was considered significant.

## 3. Results

### 3.1. Common Method Bias

This research used Harman’s single-factor test to assess common technique bias. The KMO score was 0.891, suggesting good factor analysis data. The exploratory factor analysis found eight factors (all eigenvalues >1), with the first explaining 21.69% of the variation. This fraction is much lower than the empirical criterion of 40%, indicating that common technique bias did not alter the data structure.

### 3.2. Descriptive Statistics and Correlation Analysis

In addition to the skewness and kurtosis values, Table 2 presents the performance of the two sexes in terms of the physical activity, social anxiety, and loneliness scores. Independent samples *t*-tests showed no statistically significant gender difference in the loneliness scores, but males scored significantly higher on physical activity (*p* < 0.01) and significantly lower on social anxiety than females (*p* = 0.02). The effect size analyses yielded Cohen’s *d* values of 0.402 for physical activity, 0.081 for loneliness, and 0.187 for social anxiety. Moreover, the absolute skewness and kurtosis values were below 3 and 10, respectively, indicating that the data were approximately normally distributed.

Bivariate correlation analyses of age, gender, physical activity, loneliness, and social anxiety were conducted using SPSS 27.0, and the results are detailed in Table 3. Physical activity was significantly negatively correlated with loneliness, with *r* = −0.182, *p* < 0.01, and also with social anxiety, with *r* = −0.195, *p* < 0.01. Loneliness was positively correlated with social anxiety, with *r* = 0.416, *p* < 0.01. Gender was weakly correlated with physical activity, with *r* = 0.195, *p* < 0.01, and negatively correlated with social anxiety, with *r* = −0.092, *p* < 0.05. However, there was no significant correlation between gender and loneliness, with *r* = −0.034, *p* > 0.05.

### 3.3. Moderated Mediation Model Analysis

In order to investigate both the mediating impact and the involvement of moderators, the current research used Model 7 in the Process 4.0 macro plug-in created by Hayes to perform a moderated mediation analysis. This study’s independent variable was physical activity, the mediating variable was loneliness, and the dependent variable was social anxiety. As control variables, gender and grade level were included in the model. Prior to running the model, every variable was centered. A standard 95% confidence interval was established, and the sample was repeated 5000 times to increase the analysis’s accuracy. Table 4 provides specifics on the regression analysis’s findings. The findings demonstrated that social anxiety (*β* = −0.1217, *p* < 0.001) and loneliness (*β* = −0.2160, *p* < 0.001) were substantially and adversely predicted by physical exercise. Additionally, social anxiety was substantially predicted by loneliness (*β* = 0.3937, *p* < 0.001).

**Figure 2:**
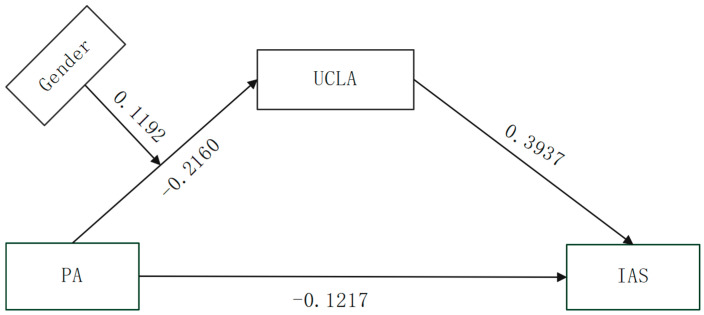
Mediation model of the effect of physical activity on social anxiety.

**Figure 3:**
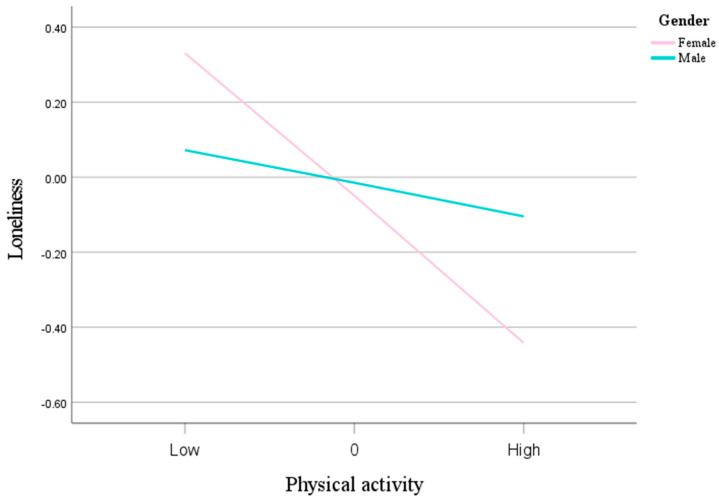
Gender as a moderator in the relationship between physical activity and loneliness.

The findings of the mediation study are extensively shown in Table 5 and Figure 2. Loneliness causes social anxiety. Furthermore, the indirect relationship between physical activity and social anxiety via loneliness was substantially reduced by gender (index = 0.1192, 95% CI: 0.0557, 0.1950). With an effect value of −0.1545 (95% CI: −0.2240, −0.0994), the mediating impact was particularly significant in the female group. In contrast, the effect value of −0.0353 (95% CI: −0.0782, −0.0039) in the male group was similarly significant. Overall, this study’s findings show that physical exercise has a direct impact on social anxiety as well as a mediating effect via loneliness, with the latter effect being more pronounced in females. This implies that the relationship between physical exercise and social anxiety may be significantly moderated by gender. As can be seen from Figure 3, the negative correlation between physical activity and loneliness is more pronounced in women than in men, further confirming the moderating effect of gender on the relationships among physical activity, loneliness, and social anxiety.

## 4. Discussion

### 4.1. Associations of Physical Activity with Loneliness and Social Anxiety

The purpose of this research was to build a moderated mediation model and examine the relationships among social anxiety, loneliness, and physical activity. This study’s findings supported research hypothesis H1 by showing a strong inverse relationship between physical exercise and social anxiety. This result is in line with earlier research by Wu et al. (2025a) and Heller et al. (2025), which supports the beneficial effects of exercise in reducing social anxiety in college students. Furthermore, this study supported research hypothesis H2 by confirming a strong negative association between loneliness and physical activity. A thorough investigation also showed that whereas greater degrees of loneliness are often correlated with higher levels of social anxiety, higher levels of physical activity are linked to lower levels of both social anxiety and loneliness. However, it is noteworthy that the correlation value, ranging from 0.195 and 0.182, is relatively low. Although statistically, physical activity negatively correlates with loneliness and social anxiety, its real-world impact is limited. So, solely relying on physical activity to ease college students’ loneliness and social anxiety may not work well; a more comprehensive strategy integrating other factors is required. Social cognitive theory (Bandura, 2001) holds that a person’s behavior is the outcome of the interplay of social, environmental, and cognitive elements. Negative self-evaluations are common among people with greater levels of social anxiety, and this may have a big impact on their behavior and motivation to be physically active. People may decide not to exercise, for instance, if they are worried about showing signs of poor body image when exercising (Brunet & Sabiston, 2009).

In addition, the association between physical activity, loneliness, and social anxiety may stem from their standard underlying links in emotion regulation. Research has shown that aerobic exercise can improve the efficiency of cognitive reappraisal by enhancing neural activity in the prefrontal cortex, facilitating functional connectivity between the amygdala and other brain regions, and increasing the plasticity of key pathways and nodes associated with emotion regulation (Wang et al., 2024). Inefficiency in cognitive reappraisal is strongly associated with social anxiety (Dryman & Heimberg, 2018), and less use of cognitive reappraisal strategies is also typical of loneliness (Preece et al., 2021). Thus, at the level of neural mechanisms, aerobic exercise supports the correlation between the three by improving emotion regulation (primarily cognitive reappraisal). Thus, engaging in certain forms of physical exercise helped college students better regulate their emotions, which in turn affected their later feelings of social anxiety and loneliness to some degree.

Meanwhile, self-determination theory (SDT) can also provide an explanation for the association between the three. According to this theory, college students experience connection, care, and support from others when they participate in physical activity, especially group exercise, which satisfies their “relatedness needs” (Zhao et al., 2024a). The fulfilment of this need, in turn, can help to reduce an individual’s loneliness and social anxiety levels. Therefore, increasing chances for team sports participation and using physical exercise as an intervention may significantly lower social anxiety and loneliness among college students.

### 4.2. Loneliness as a Key Mechanism

By developing a model that clarified the mediating function of loneliness between physical activity and social anxiety, this research not only validated the relationship between physical activity, loneliness, and social anxiety but also tested Hypothesis H3. Loneliness served as the mediating variable in this research, whereas physical activity served as the independent variable. Although this type of modeling is relatively rare in the field of physical activity, loneliness as a mediating variable has been used in other mental health studies. For example, He et al.’s (2014) study showed that social support alleviated depressive symptoms by reducing loneliness; furthermore, Maftei and Măirean (2023) also found that loneliness mediated the relationship between head-down behavior and psychological distress. Thus, loneliness often plays an important mediating role in explaining the association between behavioral factors and mental health outcomes such as social anxiety. The ability of physical activity to influence social anxiety through the mediating variable of loneliness is closely related to its own properties. The positive effects of physical activity on mental health often do not occur directly, and the mechanism of action often involves indirect pathways, such as through improved social interactions or emotional states. Loneliness, as a negative emotional state, links physical activity and subsequent mental health outcomes. The social interaction hypothesis (SIH) provides theoretical support for this phenomenon, according to which physical activity (especially social activity) can increase an individual’s social connectedness and reduce loneliness, thereby alleviating social anxiety and promoting mental health.

Furthermore, even when the mediating variable of loneliness was included, there was still a strong direct relationship between physical activity and social anxiety. This indicates that, in addition to loneliness mediating the relationship between physical activity and social anxiety, there may be other mediating variables at play. Wu et al. (2025b), in a long-term longitudinal study involving 1259 participants, noted that psychological resilience partially mediated the association between physical activity and social anxiety. Shang et al.’s (2023) study mediated by peer relations also reached similar conclusions. There are not only neural mechanisms (monoamines and endorphins) but also more complex psychosocial factors that influence college students’ participation in physical activity to improve their subsequent mental health (Peluso & de Andrade, 2005). Thus, physical activity to improve social anxiety in college students is often the result of a combination of factors.

### 4.3. The Moderating Role of Gender

The moderating influence of gender in this process was a particular focus in this research. Consistent with other results, this study found substantial disparities in physical activity levels between men and women in the early statistical analyses (Aubert et al., 2021). The gender characteristics of social anxiety and loneliness were not significantly different in the current study, in contrast to some of the earlier research (Barreto et al., 2021; Asher et al., 2017). This may be related to the specific environment of the subjects in the present study: the higher percentage of women in this environment may have promoted more female-dominated or female-participated group activities, which may have influenced the gender expression of loneliness and social anxiety.

Furthermore, in the present study, gender significantly moderated the process by which loneliness mediated social anxiety, and this indirect pathway was more significant in girls, thus confirming H4. This gap may be a result of the fact that girls are more likely to experience, or are more inclined to report, loneliness as a mediator when confronted with social anxiety. This may be related to socio-cultural factors; for example, society may be more encouraging or allowing of females expressing emotions (including loneliness), and when physical inactivity leads to social isolation, females may be more inclined to internalize social difficulties and thus experience stronger feelings of loneliness (O’Day et al., 2019). This experience of loneliness may, in turn, be more easily interpreted as a lack of social competence, which can trigger or exacerbate social anxiety. In contrast, social expectations that men “should not show weakness” may make men less willing to acknowledge or report loneliness (Gul et al., 2018). Even when physical inactivity leads to social isolation, they may suppress or otherwise express such feelings (e.g., anger, avoidance) rather than experiencing significant loneliness, and thus, the mediating effect of loneliness is not as significant as for women. In addition, according to gender theory, females may be more inclined to engage in collective, relationship-oriented physical activities that are important social interactions in their own right (Eagly & Wood, 2012). Hofstede (2011) pointed out that there are significant differences in the role positioning and value orientations of men and women in different cultures. In masculine cultures, men hold a dominant position. This type of culture emphasizes competition, achievement, and success, and it highly values material wealth and social status. In feminine cultures, however, the status of women is more equal and inclusive. This culture pays more attention to interpersonal relationships, cooperation, and emotions, regarding quality of life as an important pursuit. Moreover, sports activities precisely provide an important venue for cooperation and emotional communication. Thus, when women are physically inactive, they lose not only opportunities for exercise but also important sources of social connection and are more likely to feel isolated, which in turn affects social anxiety. Men may be more involved in competitive or individualized physical activities, which may not be as directly and strongly associated with loneliness as women. Given such differences between men and women in the relationship between physical activity and loneliness, it is of great importance to develop targeted and reasonable physical activity programs. These programs can fully take into account the characteristics of each gender, thus more effectively promoting the physical and mental health of individuals of different genders and reducing the level of social anxiety.

### 4.4. Research Limitations and Future Perspectives

Taken as a whole, this study has important theoretical and practical implications on several levels. This study built a regulated mediation model. This is the first time we examined the mediating role of loneliness between physical activity and social anxiety and the moderating effect of gender, revealing the psychological mechanisms by which physical activity affects social anxiety. Second, this research uses many theoretical approaches to describe the complicated relationship between physical activity, loneliness, and social anxiety, enriching and expanding the area of physical exercise and social anxiety.

However, this research has drawbacks. Firstly, the cross-sectional design of this research impedes causal inference. Secondly, most of the physical activity data are self-reported, which may give rise to memory bias, and it is difficult to ensure reliability and validity. Third, this study’s conclusions may not apply to other populations or areas since the sample was mostly from one city. Finally, this study only considered gender, age, and grade, without taking into account the influence of other broader demographic and psychological factors. Future research might extend as follows. First, a longitudinal strategy might better study the causal association between physical exercise and social anxiety. Second, accelerometers should be used to monitor physical activity more accurately and reliably (Troiano et al., 2008). Third, the sources and scale of the samples could be expanded, and at the same time, attention could be paid to the diversity of samples on an international scale to enhance the representativeness and generalizability of the research. Finally, the influence of a wider range of confounding factors could be considered, such as family background and past psychological conditions.

## 5. Conclusions

This study indicates that physical exercise is negatively correlated with loneliness and social anxiety. More importantly, this research has found that loneliness and gender can influence the relationship between physical activity and social anxiety among college students. These results help us understand how physical exercise impacts social anxiety and reveal gender differences. Based on these insights, the present study is expected to provide a theoretical basis for relevant interventions, such as promoting specific physical activities to alleviate the loneliness and social anxiety levels of college students, taking into account gender differences, and ultimately promoting their physical and mental health.

## Figures and Tables

**Table 1:** Distribution of subjects’ age, grade and gender.

		N	Percentage
Gender	Male	372	58.3
	Female	266	41.7
Grade	Freshman	273	42.8
	Sophomore	181	28.4
	Junior	123	19.3
	Senior	61	9.6
Age	M ± SD	19.82 ± 1.39	—

**Table 2:** Descriptive statistics.

Variable M (SD)	Total	Male (N = 372)	Female (N = 266)	*p*-Value	Var	Skew	Kurt
Age	19.81 (1.39)						
PA	20.17(20.84)	23.60 (22.35)	15.37 (17.45)	*p* < 0.01	434.25	1.494	1.811
IAS	49.42 (8.71)	48.74 (9.05)	50.37 (8.13)	*p* = 0.02	75.89	−0.137	0.532
UCLA	45.39 (8.45)	45.15 (8.64)	45.73 (8.19)	*p* = 0.312	71.41	−0.041	0.468

PA: physical activity; IAS: social anxiety; UCLA: loneliness.

**Table 3:** Pearson correlation coefficients for each variable.

	Gender	PA	UCLA	IAS
Gender	1			
PA	0.195 **	1		
UCLA	−0.034	−0.182 **	1	
IAS	−0.092 *	−0.195 **	0.416 **	1

* *p* < 0.05 ** *p* < 0.01; women: 0; men: 1.

**Table 4:** The results of the regression estimate of the chained mediation model.

Outcome Variable	Model 1UCLA				Model IAS			
	β	SE	t	*p*	β	SE	t	*p*
Age	−0.0443	0.0566	−0.7821	0.4344	0.0065	0.0523	0.1245	0.9009
Grade	0.0493	0.0567	0.8689	0.3852	−0.0428	0.0523	−0.8186	0.4133
Gender	0.0170	0.0403	0.4213	0.6737				
PA	−0.2160	0.0407	−5.3074	<0.001	−0.1217	0.0365	−2.3377	<0.001
PA × Gender	0.1494	0.0424	3.5209	<0.001				
UCLA					0.3937	0.0364	10.8098	<0.001
R^2^	0.0527				0.1890			
F	7.0326				36.8679			

**Table 5:** Conditional indirect effect for different genders.

Type	Effect	SE	Confidence Interval	
Women	−0.1545	0.0314	−0.2240	−0.0994
Men	−0.0353	0.0208	−0.0782	−0.0039
Moderated mediation index	0.1192	0.0355	0.0557	0.1950

## Data Availability

All data has been converted to specific scores and can be obtained from the following link: https://github.com/nie-ship-it/PA-IAS/blob/1c22bc0e86f99e2a7313a12749dce919ecc4c88b/PA%3B%20IAS%3B%20UCLA%20data.xlsx (Submitted on 10 May 2025, with the final version updated during the revision period).
